# Factors Associated with Acquisition of Human Infective and Animal Infective Trypanosome Infections in Domestic Livestock in Western Kenya

**DOI:** 10.1371/journal.pntd.0000941

**Published:** 2011-01-18

**Authors:** Beatrix von Wissmann, Noreen Machila, Kim Picozzi, Eric M. Fèvre, Barend M. deC. Bronsvoort, Ian G. Handel, Susan C. Welburn

**Affiliations:** 1 Centre for Infectious Diseases, School of Biomedical Sciences, College of Medicine and Veterinary Medicine, The University of Edinburgh, Edinburgh, United Kingdom; 2 School of Biological Sciences, College of Science and Engineering, The University of Edinburgh, Edinburgh, United Kingdom; 3 Epidemiology, Economics and Risk Assessment (EERA), The Roslin Institute and Royal (Dick) School of Veterinary Studies, The University of Edinburgh, Roslin, United Kingdom; IRD/CIRDES, Burkina Faso

## Abstract

**Background:**

Trypanosomiasis is regarded as a constraint on livestock production in Western Kenya where the responsibility for tsetse and trypanosomiasis control has increasingly shifted from the state to the individual livestock owner. To assess the sustainability of these localised control efforts, this study investigates biological and management risk factors associated with trypanosome infections detected by polymerase chain reaction (PCR), in a range of domestic livestock at the local scale in Busia, Kenya. Busia District also remains endemic for human sleeping sickness with sporadic cases of sleeping sickness reported.

**Results:**

In total, trypanosome infections were detected in 11.9% (329) out of the 2773 livestock sampled in Busia District. Multivariable logistic regression revealed that host species and cattle age affected overall trypanosome infection, with significantly increased odds of infection for cattle older than 18 months, and significantly lower odds of infection in pigs and small ruminants. Different grazing and watering management practices did not affect the odds of trypanosome infection, adjusted by host species. Neither anaemia nor condition score significantly affected the odds of trypanosome infection in cattle. Human infective *Trypanosoma brucei rhodesiense* were detected in 21.5% of animals infected with *T. brucei* s.l. (29/135) amounting to 1% (29/2773) of all sampled livestock, with significantly higher odds of *T. brucei rhodesiense* infections in *T. brucei* s.l. infected pigs (OR = 4.3, 95%CI 1.5-12.0) than in *T. brucei* s.l. infected cattle or small ruminants.

**Conclusions:**

Although cattle are the dominant reservoir of trypanosome infection it is unlikely that targeted treatment of only visibly diseased cattle will achieve sustainable interruption of transmission for either animal infective or zoonotic human infective trypanosomiasis, since most infections were detected in cattle that did not exhibit classical clinical signs of trypanosomiasis. Pigs were also found to be reservoirs of infection for *T. b. rhodesiense* and present a risk to local communities.

## Author Summary

Rhodesian sleeping sickness caused by *Trypanosome brucei rhodesiense* is a parasitic disease transmitted by tsetse flies which is fatal in humans if it is not treated. The parasites also infect a range of animal species in which they do not cause acute disease and may co-exist with other non human infective parasites. Busia District (Western Kenya) is a historic sleeping sickness focus. Human cases of this disease are still reported occasionally in Busia and neighbouring Teso District, most recently in 2008, showing that the human infective parasite species are still present in the area. However, trypanosomes in this region are mainly regarded as a threat to the productivity of domestic livestock and the responsibility for trypanosomiasis control has shifted from the state to livestock holders. To examine whether farmer-based control strategies can be successful, this study assessed the factors that influence trypanosomiasis in livestock at the local level.

The study showed that cattle are the livestock species most frequently affected by trypanosomes. However infection in cattle was not necessarily associated with signs of disease; furthermore pigs were shown to be important carriers of the human infective parasite. The treatment of only visibly diseased cattle to avoid losses in productivity will not successfully control the parasite in the long term. Keeping livestock in the vicinity of the homesteads also did not protect the animals from trypanosome infection. This indicated that the tsetse fly transmits the parasite in close proximity to human habitation, which could increase the risk of humans being infected.

## Introduction

Tsetse transmitted African trypanosomiasis poses a severe socio-economic impact throughout sub-Saharan Africa with losses to production estimated at over US$ 1.3 billion annually in terms of meat and milk yield in cattle [1]. Animal trypanosomiasis, is a serious constraint to productivity in Busia District in Western Province, Kenya, where there are also sporadic cases of human sleeping sickness reported [2]. An estimated 70% of the potential labour force of the district is engaged in subsistence mixed crop-livestock farming [3] in this poor rural area. Trypanosomiasis related losses include both direct livestock out-put (weight-loss, decrease in milk, decreased reproductive rate) as well as lost opportunity in terms of integration of livestock into crop production and the potential for crop-improvement (loss of draught power and manure) [1], [4]. *Trypanosoma congolense* (*T. congolense*), *T. vivax* and to a lesser extent *T. brucei* s.l. are the species that affect local African cattle in this region. Small ruminants are generally reported to be less susceptible to clinical trypanosomiasis [5], however they can harbour low grade chronic trypanosome infections, which can induce severe pathology when transmitted to cattle [6]. Pigs are moderately susceptible to *T. congolense* and *T. brucei* s.l. infections [7], [8]. *T. brucei* s.l. infections are generally less pathogenic in indigenous livestock than either *T. vivax* or *T. congolense* [9]. However in areas endemic for Rhodesian sleeping sickness, livestock play an important role as a reservoir for the human infective subspecies *T. brucei rhodesiense* (*T. b. rhodesiense*), frequently without displaying overt clinical signs of infection.

Traditionally, control of trypanosomiasis in Kenya was state-run. Until the late 1980s, large scale aerial and ground-spraying campaigns had been used by public agencies as the mainstay of tsetse and thus of trypanosomiasis control [10]. Over the last two decades, ongoing cuts in the budget of the Veterinary Department, concentrated the remaining available funds on the provision of public-goods services [11]. Trypanosomiasis was no longer perceived as an acute risk to human health in Kenya, but viewed as a livestock production disease, the control of which was in the interest of the individual livestock-owners. This shift in responsibility radically changed the scale of control efforts from area wide programmes, to small-scale community based interventions [4].

Behavioural and geographical risk factors for human sleeping sickness have been identified at the local scale in a neighbouring district in south-east Uganda (Tororo District) [12], but less is known about the epidemiology of livestock trypanosomiasis at the local scale in areas endemic for trypanosomiasis and human sleeping sickness in Kenya. This study examines the biological and management risk factors associated with trypanosomiasis infections (both animal infective and human infective, zoonotic infections), in a range of domestic livestock in Busia District, Kenya.

Making use of a unique census set of blood samples from the local livestock population in two study sites in Busia, in combination with sensitive polymerase chain reaction (PCR) technology for identification of trypanosome infections, this study aimed to establish animal inherent and management related risk factors for trypanosomiasis at the household level to identify the parasite reservoir and to aid identification of infected animals and inform control. Furthermore this study aimed to reassess the public health significance of trypanosomiasis in Busia, by investigating the presence and distribution of the human infective *T. b. rhodesiense* in the livestock reservoir as proxy for transmission risk for human sleeping sickness.

## Materials and Methods

### Ethical statement

The study used samples of blood stored in long term storage from a number of livestock species collected from the ear vein. This non invasive approach requiring minimal restraint of the animals was approved by both the University of Edinburgh Ethics Review Committee and the Kenyan Department of Veterinary Services.

### Study sites

**Figure 1:**
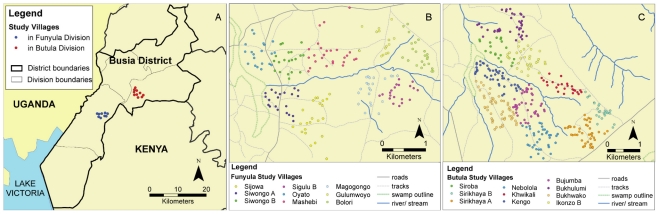
Map of sampling sites. A: overview, B: Funyula study villages, C: Butula study villages.

The study was performed in two sites within Busia District, Western Province, Kenya. Site 1 comprised nine adjacent villages and was located in Funyula Division between 0.249°–0.281° North and 34.087°–34.124° East (Datum WGS84) and Site 2 comprised ten adjacent villages and was located in Butula Division between 0.317°–0.358° North and 34.201°–34.240° East (Datum WGS84). These two sampling areas were established field sites, which were well characterised in terms of livestock-keeping dynamics and veterinary care seeking behaviour [13], [14]. Figure 1 shows a map of the study sites.

### Sampling

Census sampling targeting the entire livestock population (cattle, pigs and small ruminants) of the two sampling sites was performed in July (Funyula site) and October (Butula site) 2004, by visiting all livestock keeping homesteads in all 19 sampling villages. The geographic co-ordinates of each livestock keeping homestead, linked to a unique identification number, were recorded using a handheld global positioning system 12 (GPS 12) Personal Navigator (Garmin Ltd, Kansas, USA).

Whole blood samples from ear-veins were collected from all cattle, pigs and small ruminants at each livestock keeping homestead. Samples (100 µl) were applied to FTA Cards (Whatman, Maidstone, Kent, UK) and allowed to air dry prior to storage at room temperature [15]. A total of 2773 livestock samples from 549 livestock-keeping homesteads were collected (see table 1). Ear-vein sampling was attempted in all animals other than those below two weeks of age. In a number of animals (mainly goats and sheep) ear vein puncture failed to draw sufficient blood due to small or collapsing ear veins. Several pigs were excluded as owners were reluctant to give permission for sampling of pregnant or lactating sows and their piglets, for fear of stress causing abortion or cessation of lactation.

**Table 1:** Summary of the number of samples collected and total livestock population in sampling sites.

Livestock species	N^o^ livestock sampled			Total livestock population	Percentage of population sampled
	Funyula	Butula	total		
cattle	446	814	1260	1347	93.5%
pigs	109	203	312	495	63%
small ruminants	526	675	1201	1407	85.4%
total	1081	1692	2773	3249	85.3%

### Factors under investigation

Household identification number, animal species (cattle, pig, small ruminant) and gender (male, female) were recorded for each blood sample collected. In addition, age group, body condition score and anaemia score were recorded for all sampled cattle. Age was recorded as identified by owner or by tooth eruption pattern (category a: milk teeth, under 18 months of age; category b: one pair of permanent incisors, between 18 months and 3 years of age; category c: more than one pair of permanent incisors, over 3 years of age). Body condition score of the animal was initially scored on a scale of nine categories according to Nicholson and Butterworth (1986) [16] whereby animals are assessed as lean (L), medium (M) or fat (F), with each category subdivided into three classes, for example M-/M/M+, according to muscle mass and extent of fat deposition. Due to the low number of animals in some sub-categories, they were subsequently collapsed to the three main categories (L, M, F) for statistical analysis. Anaemia score was coded as normal (N) or anaemic (N+) as assessed by the veterinarian according to the colouration and perfusion of the mucous membranes in eyes and mouth of the animal. Anaemia score was assessed by the same experienced veterinarian throughout the whole study to exclude inter-observer bias. Anaemia scoring in cattle by visual assessment of the mucous membranes of the mouth and eyes has been previously shown to have a good correlation with blood haemoglobin levels [13].

For each homestead, the respondent was asked how many animals of each livestock species were owned by the household, and where these animals were grazed and watered. For statistical analysis, grazing and watering management for the animals of the respective species were coded into two categories (home/away): The category “*home*” was defined as animals that were fed/watered within the immediate compound of the homestead, either through grazing within the confines (usually tethered), or through feed/water being brought to the animal, whereas animals that were taken beyond the confines of the homestead compound for grazing/watering were assigned to the category “*away*”. Compounds were usually delineated by shrubbery or hedges and varied in size, with the majority of compounds being between 5 m and 20 m in diameter. When grazing and watering were analysed jointly as a combined variable (overall management) the management practice was categorised as “*home*” only when both feeding and watering practice were coded as “*home*”, otherwise the category “*away*” was assigned.

### Trypanosome characterization

All blood samples were analysed by PCR for the presence of the African animal pathogenic trypanosome species *T. brucei* s.l., *T. vivax*, *T. congolense* and *T. simiae* using two established PCR protocols on each sample. The first, the internal transcribed spacer region PCR (ITS-PCR), detects and differentiates the trypanosome species affecting livestock [17]. The second PCR was specific for *T. brucei* s.l. [18].

Any sample that was positive by at least one PCR for *T. brucei* s.l. (cumulative results for ITS-PCR and Trypanozoon specific PCR run in parallel), was considered positive for *T. brucei* s.l. and further screened in pentaplicate for the presence of the human infective subspecies *T. b. rhodesiense* using a multiplex PCR targeting the SRA gene [19] and the product visualized using southern blotting.

### Sample preparation

For each PCR reaction one 2 mm disc was cut from the samples on the FTA Card and prepared according to the manufacturer's instructions. Briefly, the discs were washed twice in FTA purification reagent to remove PCR inhibitors from the sample, followed by two washes with 1xTE buffer to remove residual FTA purification reagent. Once dried, the discs were used to seed the reactions.

### PCR reaction conditions and amplification protocols

Standard PCR amplifications were carried out in 25 µl reaction mixtures. PCR reaction conditions, primer sequences and adapted cycling conditions are shown in table 2. One positive control [genomic deoxyribonucleic acid (DNA)] and one negative control (blank FTA disc) were run with each reaction. PCR products were separated by electrophoresis in a 1.5% (w/v) agarose gel containing 0.5 µg/ml ethidium bromide and visualised by ultraviolet light.

**Table 2:** PCR primer sequences, reaction & cycling conditions and product sizes.

PCR and Primer sequence (5′ to 3′)	Specific amplicon sizes
**ITS-PCR ** [**17**]	2^nd^ Round products
1. Round (outer primers)	*T. congolense Forest*: 1501 bp
ITS1: GAT TAC GTC CCT GCC ATT TG	*T. congolense Kilifi*: 1430 bp
ITS2: TTG TTC GCT ATC GGT CTT CC	*T.congolense Savannah*: 1408 bp
	*T. congolense Tsavo*: 951 bp
2. Round (inner primers – product sizes)	*T. brucei* s.l.: 1215 bp
ITS3: GGA AGC AAA AGT CGT AAC AAG G	*T. simiae*: 847 bp
ITS4: TGT TTT CTT TTC CTC CGC TG	*T. vivax*: 620 bp
	*T. theilerie*: 998 bp
10 mM Tris-HCl, pH 9.0, 1.5 mM MgCl_2_, 50 mM KCl, 0.1% Triton X-100 and 0.01% (w/v) stabiliser (Super-Taq PCR Buffer, HT Biotechnologies, Cambridge, UK), 1 mM total dNTPs (Bioline, London, UK), 1.25 Units of Biotaq per reaction (Bioline, London, UK) and 0.2 µM of each primer; 95°C for 5 min; 35 cycles: 94°C for 60 s, 55°C for 60 s, 72° for 120 s	
**Trypanozoon ** [**18**]	
TBR1: CGA ATG AAT ATT AAA CAA TGC GCA GT	*T. brucei* s.l.: 177 bp
TBR2: AGA ACC ATT TAT TAG CTT TGT TGC	
16.0 mM (NH_4_)_2_SO_4_, 67 mM Tris-HCl (pH 8.8 at 25°C) 0.01% Tween 20 (NH_4_ Buffer, Bioline, London, UK), 1.5 mM Mg^2+^, 800 µM of total dNTPs, 0.7 Units of BIOTAQ RED^TM^ DNA Polymersase per reaction (Bioline, London, UK) and 0.4 µM of each primer; 94°C for 3 min; 30 cycles: 94°C for 60 s, 55°C for 60 s, 72°C for 30 s; final extension 72°C for 5 min	
**SRA-PLC Multiplex PCR ** [**19**]	
SRAf: GAA GAG CCC GTC AAG AAG GTT TG	SRA: *T. brucei rhodesiense*: 669 bp
SRAr: TTT TGA GCC TTC CAC AAG CTT GGG	
PLCf: CGC TTT GTT GAG GAG CTG CAA GCA	PLC: *T. brucei* s.l.: 324 bp
PLCr: TGC CAC CGC AAA GTC GTT ATT TCG	
PCR Buffer (Qiagen, Crawley, UK) containing a combination of KCl and (NH_4_)_2_SO_4_ and a final concentration of 2.5 mM MgCl_2_, 200 µM of each of the 4 dNTPs, 1.5 u of HotStarTaq DNA Polymerase (Qiagen) and 0.2 µM of each of the primers; 42 cycles: 94°C for 30 s, 63°C for 90 s, 72°C for 70 s; final extension 72°C for 10 min	

### Southern blotting using the DIG protocol

To obtain DIG-labeled probe, genomic DNA of a known *T. b. rhodesiense* stock (LIRI024) (PLC and SRA) was amplified by multiplex PCR [19]. Products were separated by electrophoresis, extracted using a MiniElute Gel Extraction Kit (Qiagen) and labeled using DIG-High Prime labeling mixture (Roche, Mannheim, Germany) according to the manufacturer's instructions. After probe yield estimation, labeled probe was used at a concentration of 25 ng/cm^3^ in hybridisation buffer, for each Southern blot.

After DNA denaturation [20 min in denaturing solution (0.5 M NaOH, 1.5 M NaCl) 20 min in neutralising solution (0.5 M Tris-HCl at pH 7.5, 3 M NaCl)], transfer of the DNA from the agarose gel onto the nitrocellulose membrane, was performed on a vacuum blotter (QBiogene, Cambridge, UK), followed by UV-cross linking to the membrane. Hybridisation with the probe and visualisation was performed according to the DIG standard protocol (Roche) provided by the manufacturer.

### Statistical analysis

Questionnaire data were recorded in a Microsoft Excel spreadsheet (Microsoft Corporation, Redmond, USA). Test results were appended to this spreadsheet and samples classified as trypanosome positive if they were positive for any of the detectable trypanosome species by either one of or both the ITS-PCR and the *T. brucei* s.l. specific PCR. Agreement between the two PCRs for the detection of *T. brucei* s.l. was assessed by calculating Cohen's Kappa coefficient and marginal homogeneity was assessed by the McNemar's test [20].

A number of risk factors were considered for entry into a multivariable logistic regression model, on the basis of their potential biological significance, including administrative division, animal species, sex, and management practice (see factors under investigation). The relationships between the factors of interest and trypanosome infection status were initially examined using univariable logistic regression. Multicollinearity of risk factors was assessed using Pearson's Chi-squared tests. Risk factors/explanatory variables were screened for each response variable. Factors with a likelihood ratio p-value of <0.2 were passed forward for inclusion in the multivariable model for each response variable.

The multivariable model was constructed by first including all variables that passed the initial screening and then dropping variables manually in a backwards elimination procedure based on the likelihood ratio test. Only variables that were significant at the 5% level in the likelihood ratio test were retained. Accurate data on age were only available for cattle and in order to allow this to be included, livestock species for cattle was further divided for each age group of cattle.

The Wald test p-values were used to compare the effect of factor levels within the variables. The potential confounding effects of those variables not retained in the final model were assessed by refitting each variable in succession into the final model and inspecting the percentage change in the odds ratio of the retained variables, with a change greater than 20% being considered evidence of confounding [20]. Administrative division was forcibly retained in the multivariable model as this variable simultaneously represented samples collected at different time points (July and October 2004) and in different study sites. A significant two-way interaction of division with the species and cattle age variable was demonstrated for the final model for overall trypanosome status, and *T. vivax* infection status, but not for the *T. brucei* s.l. model, from which division was thus ultimately dropped.

Finally the effect of the study design was taken into account by adding household as the random effect into the final model, examining the impact on the parameter estimates in the single-level model, and estimating the percentage of total variance occurring at the level of the random effect, using the latent variable approach [20]. The fit of the final fixed-effects model was assessed using the Pearson Chi-squared goodness-of-fit test, and its predictive ability was determined through the generation of receiver operator characteristics (ROC) curve. Statistical analysis was performed in R version 2.8.1 (The R foundation for Statistical Computing at http://CRAN.R-project.org).

Separate analyses were performed using *T. vivax*, *T. brucei* s.l. and *T. b. rhodesiense* status of the samples as the respective response variables. It was not possible to construct a separate multivariable logistic regression for *T. b. rhodesiense* infection status, due to few infection events.

## Results

### Samples

A total of 2773 livestock samples in 549 households (representing 85% of the targeted population) were collected in the two sampling sites (Funyula and Butula Division) in Busia District (see table 1). The total population of each livestock species was calculated from the number of animals which household respondents stated the household owned. A small fraction of livestock-keeping homesteads were excluded from sampling due to absence of the owner on sampling days (Funyula: 5/196 (2.6%); Butula 9/367 (2.5%)).

**Figure 2:**
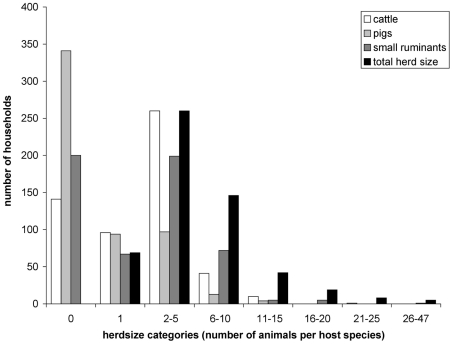
Distribution of household herdsizes. Number of households per category of herdsize for each host species and for total herdsize.

Household herdsizes ranged from one to 47 animals but in general the study area was characterized by small herdsizes with the majority of households (60%) owning five animals or less and only 5.9% of households owned over 15 animals (figure 2). Approximately three-quarters (74.3%) of livestock-owning households had cattle, close to two-thirds (63.6%) owned small ruminants and just over one third (37.9%) kept pigs (figure 2).

### Factors affecting trypanosome infection

At the univariable level, the chance of overall trypanosomiasis infection was significantly affected by the factors host species, grazing regime, overall management as well as cattle age and cattle anaemia score. The chances of infection with *T. vivax* infection or *T. brucei* s.l. infection were each significantly affected by host species, overall management, as well as cattle age. The chance of a *T. brucei* s.l. infected animal to be infected with *T. b. rhodesiense* was significantly affected by host species, with *T. b. rhodesiense* being significantly more likely to be detected in *T. brucei* s.l. infected pigs (47.4%; 9/19) than in *T. brucei* s.l. infected cattle (17.3%; 19/110).

The results of the univariable analysis of the effects of the original and combined variables on trypanosome infection status as determined by PCR in livestock are presented for overall trypanosome infection status (table 3) as well as separately for *T. vivax* (table 4), *T. brucei* s.l. (table 5), and human infective *T. b. rhodesiense* (table 6). Due to low density of infection events in sheep and goats, these livestock species were combined into the small ruminant category for data analysis. Of the variables collected only for cattle (age group, anaemia status and condition score), age group remained as the only variable with a significant effect on trypanosome infection status when a multivariable model was fitted by backward selection to the cattle data. After adjustment for cattle age, cattle anaemia status was no longer significant in the multivariable analysis and was thus not included in the final model.

**Table 3:** Univariable analysis of factors associated with overall trypanosome infection in livestock in Busia, Kenya.

Variable	Factor-levels	Total(n = 2773)	Overall trypanosome positives (%)	Odds ratio (OR)	95% confidence interval (CI)	p-value*
**Division**						
	Butula	1692	214 (12.6)	1		
	Funyula	1081	115 (10.6)	0.82	0.65–1.05	**0.11**
**Species**						**<0.001** †
	Cattle	1260	253 (20.1)	1		
	Pigs	312	36 (11.5)	0.52	0.36–0.75	<0.001
	Small ruminants	1201	40 (3.3)	0.14	0.10–0.19	<0.001
**Sex**						
	Female	2040	238 (11.7)	1		
	Male	727	91 (12.5)	1.08	0.84–1.40	**0.54**
	Missing	6	0			
**Grazing**						
	Away	666	94 (14.1)	1		
	Home	1981	219 (11.1)	0.76	0.58–0.98	**0.04** †
	Missing	126	16			
**Watering**						**0.07**
	Away	652	90 (13.8)	1		
	Home	1990	221 (11.1)	0.78	0.60–1.01	**0.07**
	Missing	131	18			
**Overall management**						
	Away	959	140 (14.6)	1		
	Home	1683	171 (10.2)	0.66	0.52–0.84	**<0.001** †
	Missing	131	18			
**Cattle age**						**<0.001** †
	a (<18 months)	407	53 (13.0)	1		
	b (18–36 months)	207	38 (18.4)	1.50	0.95–2.37	0.08
	c (>36 months)	640	161 (25.2)	2.24	1.60–3.15	<0.001
	missing	6	1			
**Cattle anaemia**						
	N (normal)	1052	199 (18.9)	1		
	N+ (anaemic)	181	46 (25.4)	1.46	1.01–2.11	**0.05** †
	missing	27	8			
**Cattle condition**						**0.38**
	Fat	70	18 (25.7)	1		
	Medium	1138	221 (19.4)	0.70	0.40–1.21	0.20
	Lean	42	10 (23.8)	0.90	0.37–2.2	0.82
	Missing	10	4			
**Species & Cattle age**						**<0.001** †
	Cattle a (<18 m)	407	53 (13)	1		
	Cattle b (18–36 m)	207	38 (18.4)	1.50	0.95–2.37	0.08
	Cattle c (>36 m)	640	161 (25.2)	2.24	1.60–3.15	<0.001
	Pigs	312	36 (11.5)	0.87	0.55–1.37	0.55
	Small ruminants	1201	40 (3.3)	0.23	0.15–0.35	<0.001
	Missing	6	1			

*Bolded p-values are likelihood ratio test p-values and non-bolded p-values are Wald test p-values.

† Likelihood ratio test p-values significant at the 5% level.

**Table 4:** Univariable analysis of factors associated with *T. vivax* infection in livestock in Busia, Kenya.

Variable	Factor-levels	Total (n = 2773)	*T. vivax* positives (%)	Odds ratio (OR)	95% confidence interval (CI)	p-value*
**Division**						
	Butula	1692	119 (7.0)	1		
	Funyula	1081	61 (5.6)	0.79	0.57–1.09	**0.14**
**Species**						**<0.001** †
	Cattle	1260	140 (11.1)	1		
	Pigs	312	12 (3.8)	0.32	0.18–0.58	<0.001
	Small ruminants	1201	28 (2.3)	0.19	0.13–0.29	<0.001
**Sex**						
	Female	2040	124 (6.1)	1		
	Male	727	56 (7.7)	1.29	0.93–1.79	**0.13**
	Missing	6	0			
**Grazing**						
	Away	666	51 (7.7)	1		
	Home	1981	115 (5.8)	0.74	0.53–1.05	**0.09**
	Missing	126	14			
**Watering**						
	Away	652	49 (7.5)	1		
	Home	1990	115 (5.8)	0.75	0.53–1.07	**0.12**
	Missing	131	16			
**Overall management**						
	Away	959	76 (7.9)	1		
	Home	1683	88 (5.2)	0.64	0.47–0.88	**0.01** †
	Missing	131	16			
**Cattle age**						**0.01** †
	a (<18 months)	407	29 (7.1)	1		
	b (18–36 months)	207	26 (12.6)	1.87	1.07–3.27	0.03
	c (>36 months)	640	84 (13.1)	1.97	1.27–3.06	0.003
	missing	6	1			
**Cattle anaemia**						
	N (normal)	1052	108 (10.3)	1		
	N+ (anaemic)	181	27 (14.9)	1.53	0.97–2.42	**0.07**
	missing	27	5			
**Cattle condition**						**0.94**
	Fat	70	7 (10.0)	1		
	Medium	1138	128 (11.2)	1.14	0.51–2.54	0.75
	Lean	42	5 (11.9)	1.22	0.36–4.11	0.75
	Missing	10	0			
**Species & Cattle age**						**<0.001** †
	Cattle a (<18 m)	407	29 (7.1)	1		
	Cattle b (18–36 m)	207	26 (12.6)	1.87	1.07–3.27	0.03
	Cattle c (>36 m)	640	84 (13.1)	1.97	1.27–3.06	0.003
	Pigs	312	12 (3.8)	0.52	0.26–1.04	0.06
	Small ruminants	1201	28 (2.3)	0.31	0.18–0.53	<0.001
	Missing	6	1			

*Bolded p-values are likelihood ratio test p-values and non-bolded p-values are Wald test p-values.

† Likelihood ratio test p-values significant at the 5% level.

**Table 5:** Univariable analysis of factors associated with *T. brucei* s.l. infection in livestock in Busia, Kenya.

Variable	Factor-levels	Total (n = 2773)	*T. brucei* s.l. positives ** (%)	Odds ratio (OR)	95% confidence interval (CI)	p-value*
**Division**						
	Butula	1692	86 (5.1)	1		
	Funyula	1081	52 (4.8)	0.94	0.66–1.34	**0.75**
**Species**						**<0.001**
	Cattle	1260	110 (8.7)	1		
	Pigs	312	19 (6.1)	0.68	0.41–1.21	0.13
	Small ruminants	1201	9 (0.7)	0.08	0.04–0.16	<0.001
**Sex**						
	Female	2040	108 (5.3)			
	Male	727	30 (4.1)	0.77	0.51–1.16	**0.21**
	Missing	6	0			
**Grazing**						
	Away	666	43 (6.5)	1		
	Home	1981	91 (4.6)	0.70	0.48–1.01	**0.06**
	Missing	126	4			
**Watering**						
	Away	652	40 (6.1)	1		
	Home	1990	94 (4.7)	0.76	0.52–1.11	**0.16**
	Missing	131	4			
**Overall management**						
	Away	959	64 (6.7)	1		
	Home	1683	70 (4.2)	0.61	0.43–0.86	**0.01**
	Missing	131	4			
**Cattle age**						**<0.001**
	a (<18 months)	407	16 (3.9)	1		
	b (18–36 months)	207	12 (5.8)	1.50	0.70–3.24	0.3
	c (>36 months)	640	82 (12.8)	3.59	2.07–6.23	<0.001
	missing	6	0			
**Cattle anaemia**						
	N (normal)	1052	89 (8.5)	1		
	N+ (anaemic)	181	19 (10.5)	1.27	0.75–2.14	**0.38**
	missing	27	2			
**Cattle condition**						**0.09**
	Fat	70	11 (15.7)	1		
	Medium	1138	90 (7.9)	0.46	0.23–0.91	0.03
	Lean	42	5 (11.9)	0.72	0.23–2.25	0.58
	Missing	10	4			
**Species & Cattle age**						**<0.001**
	Cattle a (<18 m)	407	16 (3.9)	1		
	Cattle b (18–36 m)	207	12 (5.8)	1.50	0.70–3.24	0.3
	Cattle c (>36 m)	640	82 (12.8)	3.59	2.07–6.23	<0.001
	Pigs	312	19 (6.1)	1.58	0.80–3.13	0.19
	Small ruminants	1201	9 (0.7)	0.18	0.08–0.42	<0.001
	Missing	6	0			

*Bolded p-values are likelihood ratio test p-values and non-bolded p-values are Wald test p-values.

**Cumulative detection by both ITS-PCR and species specific PCR.

**Table 6:** Univariable analysis of factors associated with *T. brucei rhodesiense* infection in livestock in Busia, Kenya.

Trypanosome species	Variable	*T. brucei* s.l. positives (n = 135**)	*T. b. rhodesiense* positives (%)	Odds ratio (OR)	95% confidence interval (CI)	p-value*
**Division**						
	Butula	86	17 (19.8)	1		
	Funyula	49	12 (24.5)	1.32	0.57–3.05	**0.52**
**Species**						**0.02** †
	Cattle	110	19 (17.3)	1		
	Pigs	19	9 (47.4)	4.31	1.54–12.04	0.01
	Small ruminants	6	1 (16.7)	0.96	0.11–8.67	0.97
**Sex**						
	Female	105	21 (20.0)	1		
	Male	30	8 (26.7)	1.45	0.57–3.72	**0.44**
**Grazing**						
	Away	42	6 (14.3)	1		
	Home	89	23 (25.8)	2.09	0.78–5.61	**0.13**
	Missing	4	0			
**Watering**						
	Away	39	7 (17.9)	1		
	Home	92	22 (23.9)	1.44	0.56–3.71	**0.45**
	Missing	4	0			
**Management**						
	Away	63	10 (15.9)	1		
	Home	68	19 (27.9)	2.06	0.87–4.85	**0.09**
	Missing	4	0			
**Cattle age**						**0.84**
	a (<18 months)	16	2 (12.5)	1		
	b (18–36 months)	12	2 (16.7)	1.40	0.17–11.68	0.76
	c (>36 months)	82	15 (18.3)	1.57	0.32–7.64	0.58
**Cattle anaemia**						
	N (normal)	89	14 (15.7)	1		
	N+ (anaemic)	19	5 (26.3)	1.91	0.59–6.16	**0.29**
	missing	2	0			
**Cattle condition**						**0.09**
	Fat	11	4 (36.4)	1		
	Medium	90	12 (13.3)	0.27	0.07–1.06	0.06
	Lean	5	2 (40.0)	1.17	0.13–10.22	0.89
	Missing	4	1			
**Species & Cattle age**						**0.1**
	Cattle a (<18 m)	16	2 (12.5)	1		
	Cattle b (18–36 m)	12	2 (16.7)	1.40	0.17–11.68	0.76
	Cattle c (>36 m)	82	15 (18.3)	1.57	0.32–7.64	0.57
	Pigs	19	9 (47.4)	6.30	1.11–35.67	0.04
	Small ruminants	6	1 (16.7)	1.40	0.10–19.01	0.80

*Bolded p-values are likelihood ratio test p-values and non-bolded p-values are Wald test p-values.

† Likelihood ratio test p-values significant at the 5% level.

**Only 135 out of 138 *T. brucei* s.l. positive samples were tested for *T. b. rhodesiense* due to insufficient sample material of the remaining three samples.

In the separate multivariable mixed-effect model for overall trypanosome infections and for *T. vivax* infections, the chance of infection was significantly affected by the combined “species and cattle age” variable, and a significant interaction effect between this factor and division was observed. Division in itself was not a significant factor. For *T. brucei* s.l. only the combined “species and cattle age” factor remained significant in the final model. The overall management variable, which was significant at the univariable level was no longer significant in any of the three final models, after adjustment for the “host species and cattle age” variable by which it was confounded.

**Figure 3:**
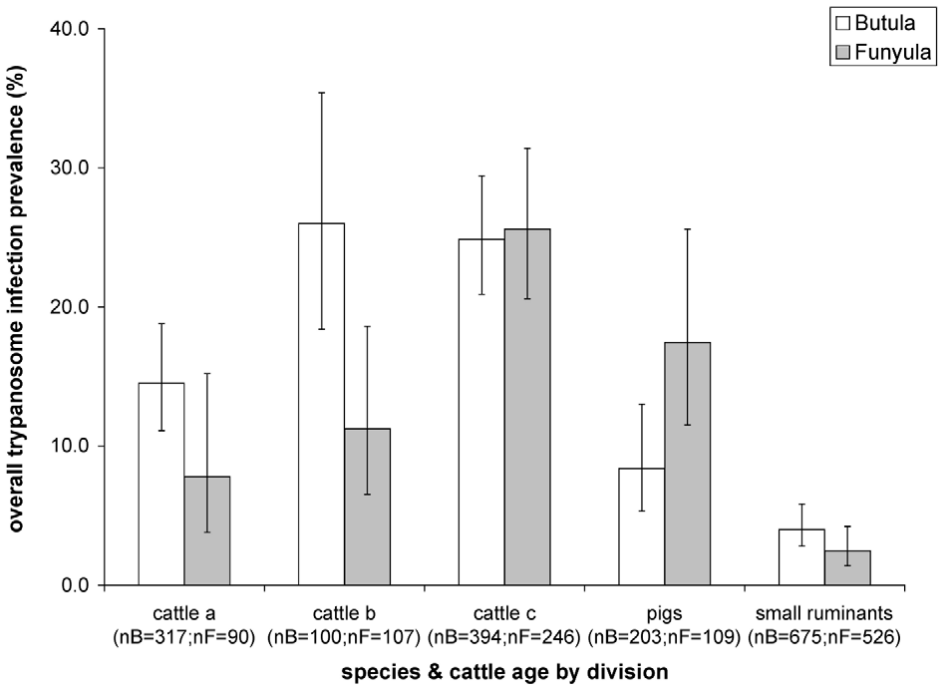
Overall trypanosomiasis prevalence by species and cattle age and division. Cattle a  =  cattle under 18 months, cattle b  =  cattle between 18 and 36 months, cattle c  =  cattle over 36 months; nB  =  number of samples from Butula site, nF  =  number of samples from Funyula site, error bars represent exact binomial 95% confidence interval.

The effect of the variables included in the final multivariable mixed-effect model constructed for overall trypanosome infection and separately for *T. brucei* s.l. and *T. vivax* are summarised in table 7. In the final model for overall trypanosomiasis, infection was significantly associated with the “species and cattle age” variable. The odds of overall trypanosome infection were significantly increased in cattle in the intermediate age group B (18–36 months) (OR = 2.11, 95% CI: 1.18–3.76) and the oldest group C (>36 months) (OR = 2.04, 95% CI:1.36–3.06) as compared to the youngest cattle age group A (<18 months), which served as the reference. The odds of overall trypanosome infection in pigs (OR = 0.51, 95% CI: 0.27–0.96) and small ruminants (OR = 0.23, 95% CI: 0.14–0.39) were significantly decreased as compared to the youngest cattle age group. Whilst division in itself did not have a significant effect on the odds of overall trypanosome infections, there was a significant interaction effect between division and the “species and cattle age” variable. This interaction effect could be attributed to a significantly lower infection prevalence detected in pigs in Butula division, as compared to Funyula Division, and some variation in the infection prevalence in the different cattle age groups, which were not statistically significant (figure 3, table 7 multivariable model). Grazing and overall management, which were significant variables at the univariable level, were confounded by the “species and cattle age” variable and were no longer significant in the multivariable analysis and therefore dropped from the final model.

**Table 7:** Multivariable mixed-effect logistic regression model of factors associated with trypanosome infection in livestock in Busia, Kenya.

Trypanosome species	Variable	Factor-level	Odds ratio (OR)	95% confidence interval (CI)	p-value*
**All tryps**	**Division**				
		Butula	1		
		Funyula	0.45	0.19–1.11	**0.22**
	**Species & Cattle age**				**<0.001** †
		CattleA (<18 m)	1		
		CattleB (18–36 m)	2.11	1.18–3.76	0.01
		Cattle C (>36 m)	2.04	1.36–3.06	<0.001
		Pigs	0.51	0.27–0.96	0.04
		Small ruminants	0.23	0.14–0.39	<0.001
	**Interaction Division:Species & Cattle age**				**<0.001** †
		Funyula:CattleB	0.74	0.23–2.43	0.62
		Funyula:CattleC	2.17	0.83–5.65	0.11
		Funyula: Pigs	5.17	1.61–16.60	0.005
		Funyula: Small ruminants	1.28	0.41–4.02	0.67
***T.vivax***	**Division**				
		Butula	1		
		Funyula	0.38	0.11–1.37	0.33
	**Species &Cattle age**				**<0.001** †
		CattleA (<18 m)	1		
		CattleB (18–36 m)	2.50	1.27–4.92	0.008
		Cattle C (>36 m)	1.57	0.93–2.65	0.09
		Pigs	0.34	0.13–0.88	0.03
		Small ruminants	0.35	0.19–0.66	0.001
	**Interaction Division:Species & Cattle age**				**0.002** †
		Funyula:CattleB	0.95	0.20–4.59	0.95
		Funyula:CattleC	3.25	0.83–12.67	0.09
		Funyula: Pigs	5.08	0.87–29.64	0.07
		Funyula: Small ruminants	1.09	0.23–5.22	0.91
***T.brucei*** ** s.l.**	**Species & Cattle age**				**<0.001** †
		CattleA (<18 m)	1		
		CattleB (18–36 m)	1.46	0.44	0.44
		Cattle C (>36 m)	4.42	<0.001	<0.001
		Pigs	1.42	0.43	0.43
		Small ruminants	0.17	<0.001	<0.001

*Bolded p-values are likelihood ratio test p-values and non-bolded p-values are Wald test p-values.

† Likelihood ratio test p-values significant at the 5% level.

In the final model fitted for *T. vivax*, the “species and cattle age” variable was significantly associated with *T. vivax* infection. With cattle in the youngest age category as the reference, the odds of *T. vivax* infection were significantly increased for cattle in the intermediate age group (OR = 2.5, 95% CI: 1.27–4.92) and significantly decreased for pigs (OR = 0.34, 95% CI: 0.13–0.88) and small ruminants (OR = 0.35, 95% CI: 0.19–0.66), however the comparative increase in odds of *T. vivax* infections in the oldest cattle age group was not statistically significant (OR = 1.57, 95% CI: 0.93–2.65). Again, division in itself did not have a significant effect on the odds of trypanosome infections, but there was a significant interaction effect between division and the “species and cattle age” variable, and division was therefore retained in the final model for *T. vivax* (table 7). Overall management, which had been significantly associated with *T. vivax* at the univariable level, was dropped from the final model, as it was no longer significant after adjustment for the “species and cattle age” variable.

Of 138 samples positive for *T. brucei* s.l. by PCR in total, 110 (79.7%) were detected by the species specific PCR [18] and 96 (69.6%) were detected using ITS-PCR [17] (table 8). Cohen's kappa test showed substantial agreement between the two PCRs (κ = 0.65; 95% CI: 0.57–0.73). A test of marginal homogeneity, testing whether the disagreement is spread evenly (McNemar's Test, χ^2^ = 2.8, df = 1, p = 0.09) was not significant, indicating that there was no significant systematic bias in the detection of *T. brucei* s.l. by either PCR method.

**Table 8:** Agreement of species specific PCR (TBR) and ITS-PCR for the detection of *T.brucei* s.l..

		ITS-PCR [17]		
		positive	negative	total
**TBR ** [**18**]	positive	68	42	110
	negative	28	2624	2652
	total	96	2677	2762*

*Agreement was tested for 2762/2773 samples, the remaining 11 samples were negative by ITS-PCR, but there was insufficient sample material to perform a separate *T. brucei* s.l. species specific PCR.

The combined “species and cattle age” variable was the only variable significantly associated with *T. brucei* s.l. infection, and was therefore the only fixed effect retained in the final model for *T. brucei* s.l. (table 7). The odds of *T. brucei* s.l. infection were significantly increased for cattle of the oldest age group C (OR = 4.42, 95% CI: 2.24–8.73), and significantly decreased for small ruminants (OR = 0.17, 95% CI: 0.06–0.47), but there was no significant difference in odds of *T. brucei* s.l. infection in the intermediate cattle age group or in pigs as compared to cattle in the youngest age group. There was no significant interaction effect with the division variable, which was therefore dropped from the final model for *T. brucei* s.l., along with the overall management variable which was no longer significant, after adjustment for the “species and cattle age” variable.

Household was included as the random effect in the final models to account for the hierarchical structure of the data. Based on the multivariable mixed-effect models fitted for overall trypanosomiasis, *T. brucei* s.l. and *T. vivax*, 11.8%, 44.8% and 7.0% of the total variance respectively occurred at the household level, as estimated using the latent variable approach [20].

The Pearson Chi-squared test statistic, calculated to assess the goodness-of-fit of the respective multivariate models, was ≪0.001 (df = 7; p>0.99) for the fixed-effects multivariable model for overall trypanosomiasis and ≪0.001 (df = 7, p>0.99) for the fixed effects multivariable model for *T. vivax*. This indicated that there was no evidence that the respective models did not fit the data well. The area under the ROC curve was 0.74 for the overall trypanosomiasis model and 0.72 for the *T. vivax* model indicating that both models have an acceptable predictive ability.

## Discussion

In the densely populated agro-pastoral study district of Busia domestic livestock are the only trypanosome reservoir of epidemiological significance. The extensive cross-sectional data set collected for the present study through census sampling, achieved coverage of over 85% of the livestock population in the sampling area permitting detailed analysis of the trypanosome infections at the household level. PCR is a sensitive molecular tool, which can increase the number of trypanosome infections detected at least two-fold when compared directly to the microscopy results from the same sample set of cattle [21], [22]. Cattle were identified as the livestock species with the highest prevalence of all trypanosome infections (20.1%), whereas the prevalence in small ruminants was low (<5%). The prevalence in pigs differed significantly between the two sampling sites, with a prevalence of 17.4% in pigs in Funyula Division as compared to only 8.4% in Butula Division (figure 3). Considering individual trypanosome species, *T. congolense* and *T. simiae* infections were detected in under 0.3% and 1.5% of all samples, respectively, and these infections were not separately analysed. Several studies conducted in Busia, have also shown low *T. congolense* infection rates of under 3% in cattle and close to 0% in small ruminants and pigs [23], [24]. In cattle and small ruminants, *T. vivax* was the most prevalent trypanosome species detected. In pigs, *T. brucei* s.l. infections predominated, with a similar prevalence to that found in cattle. There was substantial agreement for detection of *T. brucei* s.l. infections between the *T. brucei* s.l. specific PCR [18] and the ITS-PCR [17]. Both methods were employed in parallel to increase the sensitivity of detection for *T. brucei* s.l., to allow for subsequent detection of the zoonotic subspecies, *T. b. rhodesiense*.

Previous observations of a high prevalence of PCR detected trypanosomiasis in small ruminants (20–25%) in Busia District by Ng'ayo and colleagues (2005) were not observed in this study [25]. The results presented here support microscopy and PCR studies in Western Kenya and Eastern Uganda, in which cattle were identified as the most important reservoir of trypanosomiasis, low levels of infection were detected in small ruminants and highly variable infection prevalence depending on sampling sites were seen in pigs (2–20%) [8], [23], [26]. Differences in trypanosome prevalence between livestock species have previously been attributed to reduced susceptibility of small ruminants resulting in a low or transient parasitaemia [27], or lower exposure of small ruminants to tsetse bites [28]. The latter was supported by the identification of cattle and pigs as the major source of blood meals of both *Glossina fuscipes fuscipes* and *Glossina pallidipes* in this region [29]–[31].

Protection of cattle from pathogenic trypanosome infections is at the centre of productivity-motivated control strategies in this region. Trypanosome prevalence in cattle in this study was shown to significantly increase with age. A comparatively low prevalence of trypanosomiasis, observed in young cattle has previously been explained by either an inherent resistance to trypanosome infections in young animals [32], tsetse feeding preferences for adult cattle due to size and olfactory cues [33]–[35], or lower tsetse exposure of young cattle due to separate management from the rest of the herd [36]. However as animals remain infected unless treated, the higher prevalence of trypanosomiasis observed in adult cattle in the present study may simply be a result of older cattle having been exposed to tsetse for a longer time-span and thus having a higher cumulative risk of infection.

The two key indicators commonly used for the clinical diagnosis of trypanosomiasis, namely anaemia and poor body condition [37], performed poorly in the present study. There was no significant difference in trypanosome prevalence according to cattle condition score. Whilst the overall chance of a trypanosome infection was significantly increased in cattle classified as anaemic this was no longer the case after adjustment for age group and only a minority of infected animals were classified as anaemic. Over 80% of trypanosome infected cattle did not display pallor of mucous membranes. The chance of *T. brucei* s.l. infection was not significantly increased in anaemic cattle, confirming that anaemia is more commonly associated with *T. congolense* and *T. vivax* rather than *T. brucei* s.l. infections [9]. However, even the sensitivity for detecting *T. vivax* infections based on anaemia status was low in the present study, with only 20% of infected cattle being identified as anaemic by visual inspection of mucous membranes. This may either be attributed to insufficient sensitivity of visual examination of mucous membranes to detect lower grade anaemia or a certain degree of trypanotolerance in zebu cattle in Busia, resulting in sub-clinical infections. Anaemia status as classified by more elaborate methods such as packed cell volume (PCV) has been shown to be moderately sensitive for the detection of trypanosome infections, with a sensitivity of 56% being recorded using a cut-off point of PCV below 24% as indicator of anaemia [38]. However, whilst measurements of PCV or haemoglobin allow a more precise and (depending on the selected cut-off point) more sensitive determination of anaemia status, such techniques require either the use electricity (centrifuge for PCV) or fairly expensive equipment and disposables (hand held haemoglobinometer and haemocuvets), neither of which are an option for routine pen-side testing in a poor rural area such as Busia. In the present study pallor of the mucous membranes was elected as the indicator of anaemia to reflect the criteria on which veterinary clinicians or animal health workers would base their treatment decisions.

PCR will detect a significant proportion of sub-clinically infected cattle, which contribute to the reservoir of trypanosome infection in this endemic area. With very low average profit margins on livestock production in Busia [14], there is limited scope for sophisticated diagnostic procedures and block treatment of cattle. Treatment of visibly ill cattle with trypanocidal drugs, as practiced at present [13], limits immediate economic losses at the household level but is unlikely to impact on the reservoir of infections and impact on transmission of the parasite, which would be necessary for sustainable control.

Human infective *T. b. rhodesiense* were detected by PCR in a total of 19/1260 cattle (1.5%) and 9 out of the 312 pig samples (2.9%). PCR can detect sub-clinical infections with very low parasitaemia. However even low-grade infections must still be regarded as transmissible as only a single trypanosome is required to infect a tsetse fly [39] and it has been demonstrated that even during chronic, low parasiaemic phases of *T. brucei* s.l. infections in cattle, sufficient parasites are present to infect tsetse [40]. Cattle are the most important reservoir of *T. b. rhodesiense* in this region [41], [42], with up to 18% of cattle infected in an epidemic focus in Uganda [43]. The comparatively low prevalence of *T.b. rhodesiense* detected in cattle and pigs during the current study nevertheless still poses a threat to human health in this area of Western Kenya, as was demonstrated by a case of sleeping sickness reported from Busia District in early 2006 [2] and the last recorded case from neighbouring Teso District, diagnosed in 2008 (Alupe Hospital, Western Kenya, pers. comm.).

Only sporadic cases of sleeping sickness cases have been reported from Busia over the last ten years. It has been suggested that anthropogenic changes, especially increased cultivation, played a role in reducing the tsetse habitat and tsetse densities and thus reducing the overall probability of transmission to humans [44]. However, a degree of under-detection of human cases, as has been reported for Uganda [45], may also play a role in the low number of sleeping sickness cases reported from Busia.

Previous studies have demonstrated that the trypanosome prevalence detected in livestock varied significantly according to the grazing routes and type of watering places frequented. Natural river watering sites were transmission hotspots [46], [47] and cattle and small ruminants tethered for grazing within the village showed a lower probability of becoming infected [24], [26].

In the present study the majority of livestock (61%) were confined to the immediate surroundings of their respective homestead, with feed and water being provided *in situ*. There appeared to be a significant protective effect of this strategy when data were analysed at the univariable level. However, management practice was confounded by livestock species: cattle (with the highest infection prevalence) were more likely to be taken out of the compound for feeding and watering than the other livestock species, creating the impression of lower odds of infection in animals managed within the compound. Overall, data collected on management regimes in the present study did not provide evidence that confining animals within the homestead compounds decreased the likelihood of animals becoming infected. Of a total of 329 livestock samples detected to be trypanosome infected, over 50% (171) were taken from animals that did not leave the immediate vicinity of their homestead. The management practice of maintaining livestock within the immediate vicinity of the homestead rather than taking animals for grazing on communal land and watering at the river is widespread in the sampling areas of Busia, in particular for small herds. However such management appeared to provide only a very limited protective effect against trypanosome infections in livestock. Evidence of a considerable proportion of infections having been acquired by livestock maintained on homestead compounds, pointed towards an important element of transmission in the vicinity of the homestead compounds in the epidemiology of trypanosomiasis in Busia. Due to the higher probability of exposure of humans to tsetse bites, such transmission would also increase the risk of transmission of the human infective *T. b. rhodesiense* from its livestock reservoir to the human population.
